# A high-quality genome assembly of *Lactarius hatsudake* strain JH5

**DOI:** 10.1093/g3journal/jkac262

**Published:** 2022-09-28

**Authors:** Airong Shen, Chen Luo, Yun Tan, Baoming Shen, Lina Liu, Jilie Li, Zhuming Tan, Liangbin Zeng

**Affiliations:** Central South University of Forestry and Technology, Changsha 410004, China; Hunan Academy of Forestry, Changsha 410004, China; Central South University of Forestry and Technology, Changsha 410004, China; Hunan Academy of Forestry, Changsha 410004, China; Hunan Academy of Forestry, Changsha 410004, China; Hunan Academy of Forestry, Changsha 410004, China; Central South University of Forestry and Technology, Changsha 410004, China; Hunan Academy of Forestry, Changsha 410004, China; Institute of Bast Fiber Crops, Chinese Academy of Agricultural Sciences, Changsha 410205, China

**Keywords:** *Lactarius hatsudake*, Illumina, PacBio, genome

## Abstract

*Lactarius hatsudake* is a species of *Lactarius* commonly found in pine forests, is edible with a delicious and nutritious fruiting body, and exhibits medicinal properties. It is an ideal natural multifunctional food with bioactive components including fungal polysaccharides, crude fiber, unsaturated fatty acids, nucleic acid derivatives, various amino acids, and vitamins. However, biological and genomic analyses of this mycorrhizal mushroom are sparse, thereby hindering large-scale cultivation. Previously, we isolated and screened *L. hatsudake* JH5 strains and have applied our garnered knowledge to the large-scale cultivation of mycorrhizal seedlings. In this study, we produced a high-quality genome assembly of *L. hatsudake* JH5 by combining Illumina paired-end and PacBio single molecule real-time sequencing, resulting in PacBio single molecule real-time reads of 7.67 Gb and Illumina Pair-End reads of 1,560 Mb. Based on the distribution of k-mer frequencies, the genome size of this strain was estimated to be 63.84 Mb (1.14% heterozygosity). Based on de novo genome assembly, the final genome size was determined to be 76.7 Mb, with scaffold N50 of 223.2 kb and N90 of 54.5 kb, and a GC content of 54.38%. BUSCO assessment showed that genome completeness was 89.0%. The N50 length of the JH5 genome was 43.6% longer than that of the previously published *L. hatsudake* MG20 genome. This high-quality *L. hatsudake* genome assembly will facilitate research on the functional genome, molecular breeding, yield enhancement, and sustainability of *L. hatsudake* cultivation.

## Introduction

*Lactarius hatsudake* Tanaka, also known as red milk mushroom, is a high-quality wild edible and medicinal mycorrhizal fungus that is symbiotic with Pinaceae or *Quercus*. It is principally distributed in North America, Europe, and Southeast Asia within China, Korea, Thailand, and Japan (Fang *et al.* 2006). The fruiting bodies of *L. hatsudake* are nutritious and rich in bioactive components such as fungal polysaccharides, crude fiber, unsaturated fatty acids, nucleic acid derivatives, various amino acids, and vitamins (Miyazawa *et al.* 2010; Wang *et al.* 2016). Previous studies have shown that *L. hatsudake* is able to alleviate symptoms of Diabetes patients, improve immune responses, and inhibit pathogenic bacteria (Zhang *et al.* 2007; Tako *et al.* 2012), thus serving as an ideal natural multifunctional high-grade food source. *Lactarius hatsudake* is also known as cold fungus, wild goose fungus, gong fungus, silk fungus, and purple flower fungus and has become a major species in the wild edible mushroom trade in southeastern China (Li *et al.* 2018) with popularity extending to Japan, South Korea, Thailand, among other locations (Miyazawa *et al.* 2010; Tako *et al.* 2012). The mushroom can be consumed fresh, frozen, or processed into mushroom oil.

Successful cultivation of *L. hatsudake* has been a long-standing desire within production regions. Initially, *L. hatsudake*–*Pinus massoniana* Lamb was obtained by tissue isolation and mycorrhizal synthesis techniques (Tan 2005, 2006; Tan *et al.* 2007). Currently, the red milk mushroom plantation in Hunan Province covers 118 hectares with the fruiting bodies produced in 3–4 years after mycorrhizal seeding. Successful cultivation of *L. hatsudake* has led to a novel forest economy with associated ecological and economic benefits. However, due to the extended cultivation time and a need for extensive plantation management, it has been difficult to reliably achieve stable and high yields. A lack of comprehensive whole-genome analysis for *L. hatsudake* has also served to limit mechanistic analysis. What is currently known is that, based on whole-genome sequencing, sexual reproduction was found to be heterotypical coordinated in *Tuber melanosporum* (Martin *et al.* 2010), while *Laccaria bicolor* was found to possess a family of genes associated with symbiosis, called the “symbiosis toolbox” (Martin *et al.* 2008; Martin and Selosse 2008). Studies have revealed a great diversity of genomic landscapes and gene pools within Russulaceae (Lebreton *et al.* 2022). These results suggest that in-depth analysis of the genomic characteristics of mycorrhizal edible fungi by genome sequencing technology can help to identify and clarify the process of mycorrhiza formation and fruiting body development, thus contributing to an efficient and sustainable cultivation of these edible mycorrhizal fungi.

Deciphering the genome of *L. hatsudake* will provide theoretical support for the process of mycorrhiza and fruiting body formation and may guide further large-scale cultivation. Currently, 24 *Lactarius* species have undergone preliminary genomic analysis, with N50 values ranging from 5.0 to 261.3 kb (Li *et al.* 2018; Lebreton *et al.* 2022). For *L. hatsudake*, using Illumina sequencing technology, Li *et al.* (2018) reported a draft genome of *L. hatsudake* MG20 with an N50 of 5.0 kb. More recently, using PacBio sequencing platform, Lebreton *et al.* (2022) published the genome assembly of *L. hatsudake* 109 with an N50 of 261.3 kb.

Previously, we isolated and screened the JH5 strain of *L. hatsudake* from the field, which has been used for large-scale culture of mycorrhizal seedlings. This strain exhibited rapid growth, adaptation to various nutritional environments, high mycorrhizal infection rate, high competitiveness, and genetic stability (Tan *et al.* 2021). Herein, we performed de novo sequencing of JH5 genome by combining the Illumina and PacBio platform, and provided an improved genome assembly of *L. hatsudake*. This study aims to develop foundational genomic resource for gene functional studies as well as molecular breeding of *L. hatsudake* to improve yield.

## Materials and methods

### Fungal strain and DNA extraction

*Lactarius hatsudake* strain JH5 was obtained from the production and promotion base established by the project team in Pumen Village, Jiahe County, Chenzhou City, Hunan Province. *Lactarius hatsudake* JH5 was selected which was stored in the General Microbiology Center of China microbial species Preservation and Administration Committee, registration number CGMCCNo19369*.* Mycelia of JH5 was grown in 100-ml biotin-aneurine-folic acid liquid medium at 22°C, 120 rpm for 14 days in darkness. Then JH5 mycelia were separated from the culture medium, frozen in liquid nitrogen and ground to a fine powder and subjected to genome sequencing. DNA was extracted using the DNA extraction kit from TIANGEN, Beijing, China, and the purity and integrity of genomic DNA was determined by agarose gel electrophoresis.

### Genome sequencing and assembly

The genome of *L. hatsudake* JH5 was de novo sequenced using high-throughput Illumina Hiseq X-Ten and PacBio RSII long-read sequencing platforms (PacBio P6-C4) at Beijing Novogene Technology. DNA libraries with 350-bp inserts were constructed and sequenced on the Illumina HiseqX-Ten platform. For the PacBio RSII platform, a 20-kb library was generated and sequenced. The genome size of *L. hatsudake* JH5 was estimated by the k-mer method using sequencing data from the Illumina DNA library. A 15-mer frequency distribution analysis of the quality-filtered reads was performed using Jellyfish v2.2.10 (Marcais and Kingsford 2011). Genome size, heterozygosity, and repeat content were then estimated by the Genome Scope web tool (Vurture *et al.* 2017).

The genome of JH5 was de novo assembled in 3 steps. Assembly of contigs was performed with FALCON (version 0.7.0; Chin *et al.* 2016). In brief, the longest 40× reads were selected as “seed” reads for error correction (“pre-assembly”). Preassembly in FALCON uses DALigner to perform all-by-all alignments of the raw reads. The FALCON assembly resulted in 312 primary contigs. The initial polishing was performed with Arrow (included in the FALCON-Unzip) exclusively using PacBio (https://www.pacb.com/support/software-downloads/) long reads, and then SSPACE-LongReads was implemented to scaffold the contigs. Finally, Pilon (v 1.23; Walker *et al.* 2014) was utilized to further correct the PacBio-corrected contigs with accurate Illumina short reads and generate the genome assembly of *L. hatsudake* JH5. The completeness of the JH5 genome assembly was evaluated using BUSCO 3.1.0 (Benchmarking Universal Single-Copy Orthologs) with comparison to lineage dataset fungi_odb9 (Creation date: 2016 October 21, number of species: 85, number of BUSCOs: 290; Simao *et al.* 2015).

### Genome annotation

To annotate the assembled JH5 genome, we used funannotate (v1.5.2; Love *et al.* 2019) with the pipeline described in https://funannotate.readthedocs.io/en/latest/tutorials.html with the following commands: funannotate mask, to softmask the genome, funannotate training, and funannotate predict to generate preliminary gene models, and consensus gene models [using: AUGUSTUS (Stanke and Waack 2003), GeneMark (Borodovsky and McIninch 1993), and EVidenceModeler (Haas *et al.* 2008)], and funannotate annotate to add functional annotation, in addition, protein-coding gene (PCG) models also were identified according to our 12 transcriptome data (PRJNA841037). The rRNA was predicted by using RNAmmer v1.2 (Lagesen *et al.* 2007), and tRNAs were identified with tRNAscan-SE v1.4 (Lowe and Eddy 1997). The sRNA was identified by comparing with the Rfam database (Gardner *et al.* 2009). The functional annotation obtained with funannotate includes Interpro terms, Pfam domains, CAZYmes (CAZY_DB: 201604), secreted proteins, proteases (MEROPS), BUSCO groups, Eggnog annotations, Clusters of Orthologous Groups (COGs), GO ontology, secretion of signal peptides, and transmembrane domains (the full annotation is available in Supplementary Table 1).

## Results and discussion

### High-quality genome assembly of *L. hatsudake* JH5

**Fig. 1.**
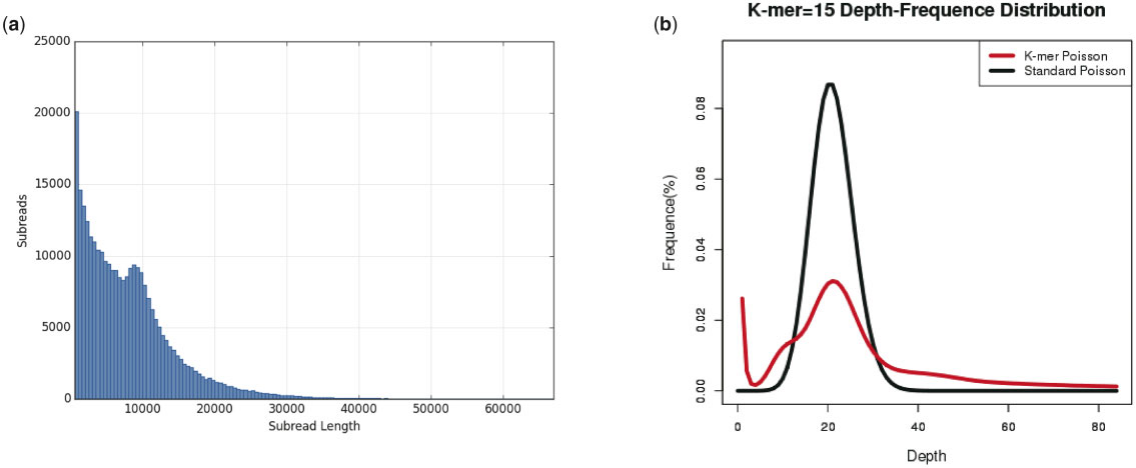
Read statistics and the distribution of K-mers. a) The sequence read length distribution map of each sample library (the abscissa represents sequence read length, and the ordinate represents the number of reads corresponding to sequence read length). b) Sample 15-mer statistical figure (the abscissa indicates the kmer depth, the ordinate indicates the proportion of the frequencies at each depth to the total number of the frequency. The red curve is the 15-mer depth distribution curve of the sequencing data, and the black curve is the standard Poisson distribution curve closest to it).

**Fig. 2.**
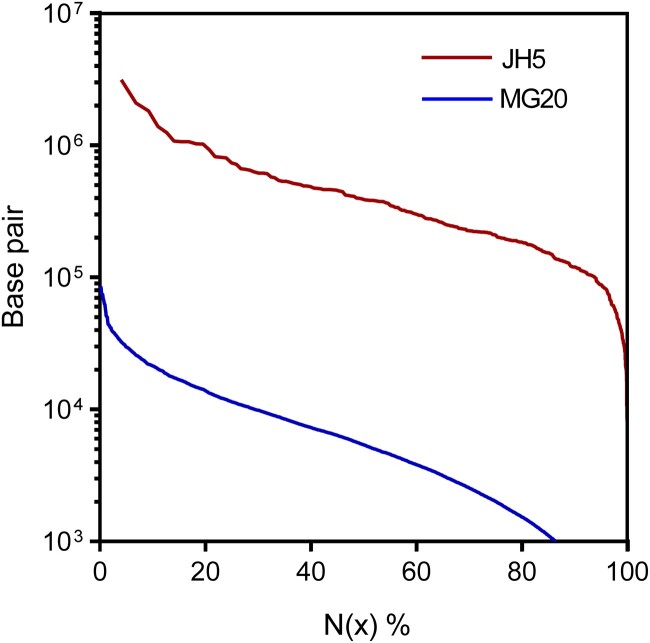
Comparison of length of Scaffold N(x) distribution between *L. hatsudake* JH5 and MG20.

To construct a high-quality reference genome of *L. hatsudake* JH5, a total of 7.67 Gb PacBio single molecule real-time (SMRT) reads and 1,560 Mb Illumina pair-end reads were generated in this study. The PacBio read lengths ranged from 200 to 50,000 bp with an average read length of 7,418 bp (Fig. 1a). We estimated the genome size of *L. hatsudake* JH5 as 63.84 Mb with a heterozygosity rate of 1.14% via the distribution of k-mer frequency using Illumina paired-end (PE) reads (Fig. 1b). High-quality PacBio SMRT reads were used to assemble the *L. hatsudake* genome. The contigs were then polished using Illumina PE reads, which yielded a draft genome assembly of 76.7 Mb, with contig N50 of 223.2 kb, N90 of 54.5 kb, GC content of 54.4%, and a BUSCO result of 89.0% (Table 1). We annotated 19,616 genes with an average gene length of 1,765 bp. The cumulative length of genes was 34.6 Mb, which accounted for 45.14% of the whole JH5 genome (Tables 1 and 2). The size of contig N50 and BUSCO results was higher than that of the previously published *L. hatsudake* MG20 genome (contig N50:5,268 bp, BUSCO result: 84.5%; Tables 1 and 2). Compared with the N50 obtained only by Illumina sequencing, the N50 length was 44.6 times higher and the number of genes increased by 1.06 times. The Scaffold N(x) length distribution in *L. hatsudake* JH5 was also significantly higher than the length of Scaffold N(x) distribution in *L. hatsudake* MG20 (Fig. 2). In total, this *L. hatsudake* genome assembly represents a significant improvement than that of other previously released *Lactarius* genomes (contig N50: 5.0–261.3 kb; Table 1; Saier *et al.* 2014; Li *et al.* 2018; Lebreton *et al.* 2022). Compared with *L. hatsudake* 109 genome assembly, JH5 genome show the considerable N50 length (223.2 kb vs 261.3 kb), and BUSCO results (89.0% vs 87.6*%*). However, the number of scaffolds of JH5 genome was far fewer than that of *L. hatsudake* 109 genome (312 vs 815), which indicated JH5 genome presented here was less fragmented. Furthermore, 2,785 additional genes were predicted compared to *L. hatsudake* 109 genome (Tables 1 and 2).

**Table 1.** BUSCO evaluation comparison of *Lactarius* spp.

Species	Scaffold N50 (kb)	Scaffold total (Mb)	Gene number	BUSCO results (%)
*L. rugatus* (MG108)^a^	5.4(S)	38.6(S)	12,848	79.7
*L. hatsudake* (MG20)^a^	5.0(S)	73.2(S)	18,513	84.5
*L. hygrophoroides* (MG19)^a^	11.1(S)	54.5(S)	14,415	86.9
*L. trivialis* (MG71)^a^	33.8(S)	35.3(S)	13,584	91.0
*L. deliciosus* (MG9)^a^	19.1(S)	54.2(S)	12,997	90.6
*L. echinatus* (MG122)^a^	11.7(S)	47.1(S)	13,477	87.9
*L. indigo* (MG109)^a^	12.8(S)	76.3(S)	21,539	88.6
*Lactarius*. sp (MG121)^a^	12.1(S)	63.7(S)	23,531	89.3
*L. pinguis* (MG27)^a^	18.3(S)	47.4(S)	15,740	84.2
*L. piperatus* (MG49)^a^	15.7(S)	49.1(S)	16,161	82.1
*L. rugatus* (MG108)^a^	5.4(S)	38.6(S)	12,848	79.7
*L. trivialis* (MG71)^a^	33.8(S)	35.3(S)	13,584	91.0
*L. volemus* (MG8)^a^	26.8(S)	42.4(S)	13,942	91.1
*Lactarius* sp (MG50)^a^	12.0(S)	64.9(S)	18,611	85.5
*L. hatsudake* (109)^b^	261.3(C)	95.5(C)	16,831	87.6
*L. akahatsu^b^*	249.0(C)	80.3(C)	12,853	92.1
*L. deliciosus^b^*	318.6(C)	96.0(C)	18,193	84.2
*L. hengduanensis^b^*	72.5(C)	62.0(C)	15,957	89.4
*L. psammicola^b^*	110.0(C)	69.8(C)	13,442	91.4
*L. pseudohatsudake^b^*	149.4(C)	99.7(C)	20,824	88.0
*L quietus^b^*	89.8(C)	115.9(C)	18,943	88.1
*L. sanguifluus^b^*	235.6(C)	86.2(C)	17,481	86.6
*L. vividus^b^*	202.6(C)	69.0(C)	11,612	95.8
*L. hatsudake* (JH5) (in this study)	223.2(C)	76.7(C)	19,616	89.0

a The data are quoted from Li *et al.* (2018).

b The data are quoted from Lebreton *et al.* (2022), S: Scaffold, N50 length obtained by Illumina sequencing platform, C: contig, N50 length obtained by PacBio sequencing platform.

**Table 2.** Comparison of JH5, MG20, and 109 genome assembly and annotation.

Sample ID	JH5	MG20	109
Scaffolds	312	36,963	815
Contigs	312	37,440	815
Max length of contigs (bp)	3,148,306	85,124	1,836,981
N50 length of contigs (bp)	223,180	5,268	261,250
N90 length of contigs (bp)	54,521	519	51,284
Total length (Mb)	76.7	73.8	95.5
GC (%)	54.4	51.9	52.1
Gene number (#)	19,616	18,513	16831
Gene total length (Mb)	34.6	19.0	29.0
Gene average length (bp)	1,765	1,025	1,724
Gene length/genome (%)	45.1	26.0	30.4

### Identification of repetitive sequences

In this study, a total of 33,787 repeat sequences were predicted within the *L. hatsudake* JH5 genome. Among these, the number of long terminal repeat sequences was 12,436, which accounted for 10.18%, at an average length of 651 bp. This was followed by tandem repeat sequences (TR), which consisted of 10,801 sequences (1.57% of total bases), long interspersed nuclear elements (0.19%), short interspersed nuclear elements (0.13%), minisatellite DNA (0.57%), and microsatellite DNA (0.09%) (Table 3).

**Table 3.** Prediction of JH5 repeat sequences of samples.

Type	Number (#)	Total length (bp)	In genome (%)	Average length (bp)
LTR	12,436	7,806,839	10.1769	651
DNA	1,065	146,746	0.1913	142
LINE	912	98,384	0.1283	116
SINE	120	7,861	0.0102	67
TR	10,801	1,201,033	1.5656	112
Minisa-ellite DNA	7,224	439,810	0.5733	61
Microsatellite DNA	1,229	68,531	0.0893	56

Note: LTR: Long Terminal Repeat sequences; DNA: DNA transposon; LINE: Long interspersed nuclear elements; SINE: Short interspersed nuclear elements; TR: Tandem Repeat; Mini satellite DNA: microsatellite DNA; Microsatellite DNA: microsatellite.

### Identification of noncoding RNAs

Noncoding RNAs are a type of RNA that has been found to perform a variety of biological functions. It does not carry information that is translated into proteins though it still directly plays a role in activities at the RNA level (Bracher *et al.* 2020). Among microbes, sRNA, rRNA, and tRNA are the most commonly studied. For *L. hatsudake* JH5, tRNAs were found to be the most abundant, with a total length of 15.97 kb. This was followed by 5S rRNA, 18 and 28 s, for a total of 9, with a total length of 8.0 kb. In addition, there were 19 snRNAs, with an average length of 121 bp and a total length of 2.3 kb (Table 4).

**Table 4.** Noncoding RNA statistical results after de-redundancy.

Type	Number (#)	Average length (bp)	Total length (kb)
tRNA	204	78	16.0
5s (de novo)	6	114	0.7
18s (de novo)	2	1,644	3.3
28s (de novo)	1	3,966	4.0
snRNA	19	121	2.3

tRNA, transfer RNA; snRNA, small nuclear RNA; rRNA, ribosomal RNA.

**Table 5.** Comparison of CAZy functional classification in *Lactarius* spp. and several other basidiomycetes.

Sample ID	CBM	CE	GH	GT	PL	AA	TOTAL
*L. hatsudake* (JH5)	24	12	110	69	1	50	266
*L. hatsudake* (MG20)^a^	12	10	27	12	2	9	72
*L. deliciosus* (MG9)^a^	9	12	34	16	1	9	81
*L. echinatus* (MG122)^a^	11	10	23	20	2	17	83
*L. hygrophoroides* (MG19)^a^	9	9	24	16	0	14	72
*L. indigo* (MG109)^a^	11	9	30	29	3	14	96
*L. pinguis* (MG27)^a^	5	16	26	18	0	17	82
*L. piperatus* (MG49)^a^	7	10	23	13	0	16	69
*L. rugatus* (MG108)^a^	1	9	18	12	2	10	52
*L. svolemus* (MG8)^a^	5	13	26	19	2	15	80
*Boletus edulis* (MG6)^a^	12	16	34	13	2	14	91
*T. calosporum* (MG102)^a^	9	13	22	19	1	13	77
*L. edodes* ^b^	58	31	245	75	9	85	461
*P. chrysosporium* ^b^	61	26	182	68	6	97	397
*P. ostreatus* ^b^	85	32	231	65	23	131	521
*H. erinaceus* ^b^	4	26	161	59	7	84	341

a The data is quoted from Li *et al.* 2020.

b The data is quoted from Gong *et al.* 2020.

### Gene function analysis

**Fig. 3.**
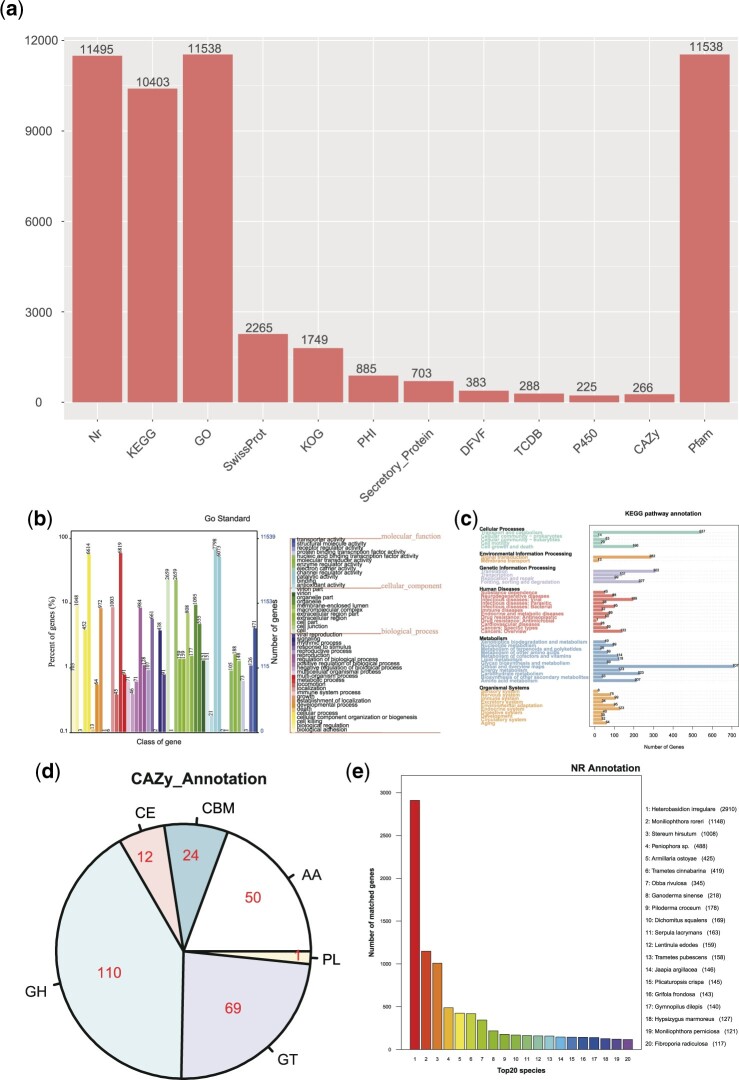
Gene annotation of *L. hatsudake* JH5. a) Statistical chart of the results of gene function analysis (the columns in the figure represent the number of genes annotated by the coding genes in each database). b) Gene functional annotation based on the GO functional classification map (the abscissa represents the GO functional classification on the sample annotation, the right ordinate indicates the number of genes on the annotation, and the left ordinate indicates the percentage of the number of genes in the annotation as a percentage of all encoded genes). c) Gene function annotation based on the KEGG metabolic pathway classification map (the number on the bar chart represents the number of genes on the annotation; the other axis is the code of each functional class of level 1 in the database, the code is explained in the corresponding illustration). d) CAZy functional classification and corresponding gene quantity map. 3E: NR database species annotation statistical chart (Abscissa represents species ID, ordinate indicates the number of genes on the annotation).

The EVM pipeline was used to predict the PCGs of the *L. hatsudake* JH5 genome, with a total of 19,616 gene models identified and 14,261 (72.70%) were expressed PCG models based on the transcriptome data. According to gene function analysis, all of the predicted genes were annotated using 11 databases (Fig. 3a), including NR, KEGG, GO, etc. Among the NR databases, the KEGG, GO, and Pfam databases produced 11,495 annotations (22.20%). The KEGG database annotated 10,403 (20.09%) while GO and Pfam results were the same at 11,538 annotations (22.28%). The SwissProt and KOG database annotated 2,265 and 1,794 genes, accounting for 4.37% and 3.46%, respectively. Lastly, the P450 and CAZy databases had the lowest number of genes annotated, accounting for 0.43% and 0.51%, respectively.

### GO database annotation

The GO database was used to interpret annotated genes across 3 aspects: Cellular components, molecular functions, and biological processes. A total of 11,538 genes were annotated to the GO database, with cellular components accounting for 19.74%, molecular functions for 34.57%, and biological processes for 45.68%. Among these, the number of relationships to “binding” was the largest, reaching 7,798, followed by the metabolic process at 6,819, cell transformation at 6,614, and “catalytic activity” at 6,073 (Fig. 3b).

### KEGG database annotation

The KEGG database was implemented in order to annotate genes covering the aspects of cellular process, environmental information processing, genetic information processing, human diseases, metabolism and organismal systems. Metabolism accounted for the largest proportion (35.20%) while the number of genes annotated to transport and catabolism was maximum, followed by human diseases (16.20%) with the largest number of annotated genes annotated as “Viral” (Fig. 3c).

### Carbohydrate enzymes database annotation

A database of carbohydrate enzymes was used that included a family of enzymes that can catalyze carbohydrate degradation, modification, and biosynthesis within 5 principal categories: glycoside hydrolases (GHs), glycosyl transferases (GTs), polysaccharide lyases (PLs), carbohydrate esterases (CEs), and auxiliary activities (AAs). The GH family comprised the largest proportion, with 110 annotated genes accounting for 41.35% (Fig. 3d). The GT family annotated 69 genes that contained 25.94% but the PL family annotated only one family that comprised only 0.38%. The largest abundances within the GH family indicates that JH5 plays an important role in the formation of monosaccharides, oligosaccharides, or carbohydrate complexes, the synthesis of alkyl glycosides and aromatic glycosides, the glycosylation of amino acids and peptides, and the glycosylation of antibiotics (Fig. 3d). The comparison of CAZy functional classification in *Lactarius* spp. showed that the annotated gene numbers of JH5 were higher than other *Lactarius* spp. (Table 4). We also found that the number of carbohydrate enzymes of *L. hatsudake* JH5 was lower than saprophytic fungi such as *Lentinula edodes*, *Phanerochaete chrysosporium*, *Pleurotus ostreatus*, and *Hericium erinaceus*. The results showed that perhaps due to the nature of JH5 as symbiotic mycorrhizal fungi, there were few CAZymes identified in *Lactarius*, with the capability of carbohydrate degradation weaker than that of white rot fungi. However, the numbers of GH and GT families in *L. hatsudake* JH5 were higher than other edible mycorrhizal fungi such as *L. hatsudake* MG20, other *Lactarius* spp., *Tuber calosporum* and *Boletus edulis*. As such, it appears that the capability of carbohydrate degradation is stronger than that of other mycorrhizal fungi of *Lactarius*.

### NR database annotation

The annotation of the NR database resulted in a total of 11,495 genes, of which JH5 exhibited the highest similarity with *Heterobasidion irregulare*. Here, 2,910 genes were annotated to the species for a total of 25.36% of all genes. This was followed by similarity to *Moniliophthora roreri* and *Stereum hirsutum*, with 1,148 (9.99%) and 1,008 (8.77%) genes shared, respectively, *Fibroporia radiculosa*, with 117 genes. Of these most close genomic relatives, *H. irregulare* is known to cause Korean pine root rot (Blanchette *et al.* 2015), *S. hirsutum* is a crustal fungus that can secrete “red latex glue” (Kuo2008), *Obba rivulosa* can secrete “sap” (Mainardi *et al.* 2018; Kontro *et al.* 2020), and *Peniophora* sp. is capable of producing lactose. These data indicate that the majority of the genetic annotations of *L. hatsudake* strain JH5 are related to “mycorrhiza” and “red juice.” Through comparison of the NR database, this genome was found to exhibit a high degree of similarity with other genomes, likely since the species are closely related and genes have not undergone major sequence differentiation (Fig. 3e).

### Conclusion

In order to improve the genome assembly of *L. hatsudake*, we performed de novo sequencing and assembly of *L. hatsudake* JH5 by combining Illumina and PacBio sequencing. A total sequence length of 76.7 Mb of JH5 genome was assembled into 312 scaffolds with an N50 of 223.2 kb, and encoded 19,616 putative predicted genes. Compared with the released *Lactarius* spp. genomes, JH5 genome assembly presented the improved completeness and integrity. Here, the high-quality genome assembly of JH5 provides important insights into the biology of *L. hatsudake*. In addition, the identified genes may enhance our understanding of predicted gene function, enabling the study of biosynthesis of active compounds. Further research could focus on genes associated with growth and development or the biosynthesis of secondary metabolites. Although incomplete, the basic information provided by the elucidation of the *L. hatsudake* genome in this study is a novel attempt to facilitate biologically and agriculturally based research and thus support future applications of fungal species.

## Supplementary Material

jkac262_Supplementary_Table_S1

## Data Availability

Sequencing data and genome assembly of JH5 for this project have been deposited in NCBI databases under project accession number PRJNA605941 (https://www.ncbi.nlm.nih.gov/bioproject/PRJNA841037, 2022/10/01). Transcriptome data of JH5 were deposited into GenBank under the accession numbers of PRJNA841037 (https://www.ncbi.nlm.nih.gov/bioproject/PRJNA605941, 2022/10/01). Supplemental material is available at G3 online.
